# New Technique of Reverse Bone Grafting With Core Decompression and Enriching With Regenerative Medicine Techniques for Grade 2 and Grade 3 Avascular Necrosis of Both Hips

**DOI:** 10.7759/cureus.51425

**Published:** 2023-12-31

**Authors:** Saksham Goyal, Sandeep Shrivastav, Ratnakar Ambade, Aditya Pundkar, Ashutosh Lohiya

**Affiliations:** 1 Department of Orthopaedics, Jawaharlal Nehru Medical College, Datta Meghe Institute of Higher Education and Research, Wardha, IND

**Keywords:** platelet-rich plasma (prp), bone marrow aspirate concentrate (bmac), core decompression, regenerative medicine, avascular necrosis

## Abstract

Early avascular necrosis (AVN) of the hip poses a significant clinical challenge, requiring prompt recognition and intervention to mitigate long-term complications. A case report describing a 30-year-old man with bilateral hip AVN is presented here. In addition, to reverse bone grafting and core decompression of both hips, the patient had platelet-rich plasma (PRP) infiltration in the right hip and bone marrow aspirate concentrate (BMAC) infiltration in the left hip. This method attempted to stop the disease's development and promote hip regeneration in both. Significant pain reduction and postoperative functional gains in both hips are seen in this instance. These results highlight the potential of combined orthopedic and regenerative therapies in young individuals with hip AVN and highlight the necessity of early intervention for maintaining long-term hip function.

## Introduction

The loss of subchondral bone structure is a result of aberrant microcirculation resulting from femoral head osteonecrosis. Although the underlying pathophysiology is unclear, risk factors probably have some effect on microcirculation, albeit research has not shown this. Necrosis and aberrant microcirculation are the typical endpoints. The ensuing collapse of the subchondral bone causes progressive secondary arthritis [1,2]. While avascular necrosis (AVN) predominantly afflicts elderly individuals and is frequently associated with chronic medical conditions and long-term corticosteroid use, it can also affect younger individuals. In the younger population, AVN is frequently connected to trauma, excessive consumption of alcohol, and certain illnesses such as sickle cell disease or systemic lupus erythematosus. It has also been connected to COVID-19 more recently. Many studies are being conducted, but they are yet to produce meaningful results [3]. The best course of action necessitates protecting the femoral head or at the very least prolonging its collapse or the development of degenerative alterations. AVN is the cause of five to 12 percent of total hip replacement surgeries [4]. AVN is linked to several atraumatic and traumatic disorders [5]. The disease progresses naturally, with subchondral fractures leading to collapse and osteoarthritis [6,7]. Depending on the disease's stage, Mont and Hungerford's meta-analysis of the natural history revealed that the femoral head was maintained in 13% to 35% of hips [4]. There is an ongoing debate on the non-prosthetic management of AVN. Multiple options have been discussed, such as different osteotomies, vascularized and non-vascularized bone grafting, and core decompression.

This case study explores the peculiar case of a male patient, 30 years old, who was diagnosed with bilateral AVN of the hips, a rare ailment for this age range, but recently the number of cases has been on a rising trend. Since the hips are essential for ambulation and daily activities, the ailment has a considerable negative impact on the patient's quality of life. Maintaining hip function while averting long-term additional decline is difficult when controlling AVN in young adults. AVN is often treated with a focus on reducing discomfort, regaining joint function, and stopping the disease's development. The primary choices have been surgical operations such as joint replacement and core decompression. Nevertheless, studies have indicated that the overall clinical success rate of core decompression is just 63.5%, and the success rate of hip salvage or total hip replacement (THR) surgery that follows is only around 33% [8]. Because of this, the procedure's use has been contested. To improve core decompression outcomes, various regeneration procedures have recently been suggested as a way to treat the early stages of AVN [9].

In this case, the patient underwent reverse bone grafting, a procedure that involves replacing necrotic bone in the same location with healthy bone. This was combined with core decompression for both hips. Additionally, to explore the potential for regeneration, bone marrow aspirate concentrate (BMAC) and platelet-rich plasma (PRP) were injected into the left hip, while only PRP was administered into the right hip. The goals of the combined orthopedic and regenerative medicine strategy were to reduce hip discomfort, slow the course of AVN, and encourage hip tissue regeneration. This case study presents a comprehensive strategy for managing AVN in a patient who is a young adult. The goal of the multidisciplinary treatment plan is to provide long-term solutions that maintain hip function and enhance the patient's overall quality of life, in addition to treating the acute symptoms. The clinical presentation of the patient, the surgical and regenerative methods used, and the postoperative results are covered in depth in the parts that follow, with an emphasis on the potential advantages of such therapies in young people with bilateral hip pain.

## Case presentation

A 30-year-old male presented to our orthopedic clinic with a chief complaint of progressive left hip pain and limited mobility with moderate pain over the right hip. He reported that the pain had been ongoing for several months and was unresponsive to conservative treatments, including rest and pain medications. Of note, the patient had no significant past medical history or medication use, which might have predisposed him to AVN. Upon examination, the patient displayed pain with passive and active hip movement. The range of motion was significantly reduced in the left hip and moderately reduced in the right hip, and there was evident discomfort during the examination. The patient experienced particular discomfort during internal and external rotation of the hips. The movements were: flexion - 55 degrees, external rotation - 30 degrees, internal rotation - 10 degrees, abduction - 25 degrees, and adduction - 15 degrees over the left hip.

**Figure 1:**
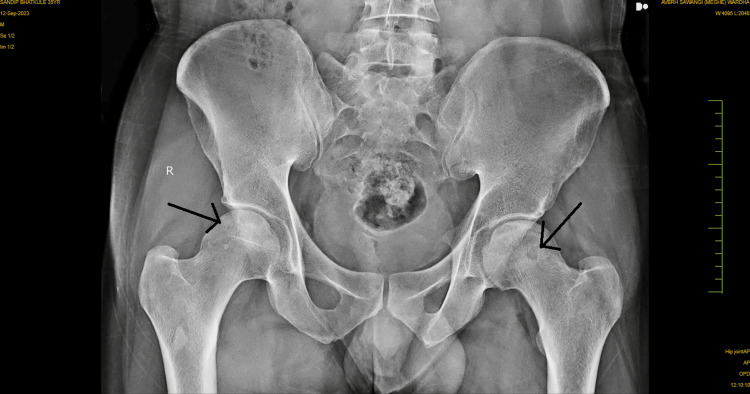
Anteroposterior radiograph of both hips showing cysts on both hips

**Figure 2:**
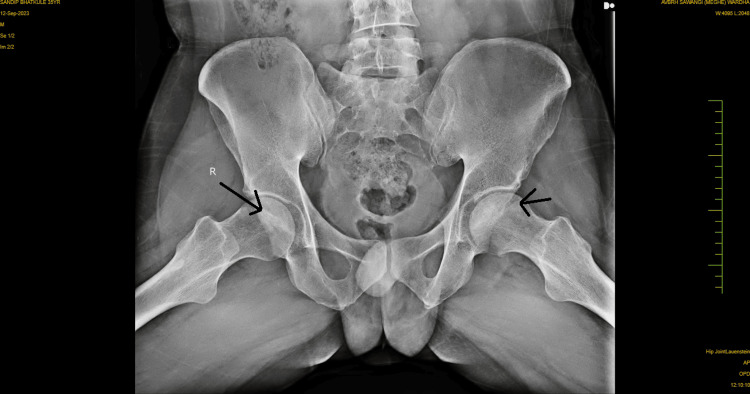
Lateral radiograph of both hips showing cysts on both hips

**Figure 3:**
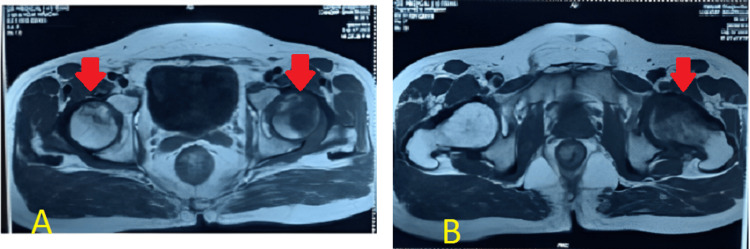
Axial section (A) and (B) of MRI, both hips showing more destruction on the left side as compared to the right The axial slices of images A and B show avascular necrosis, or the loss of vascularity, on both sides, with the left side showing more of it.

**Figure 4:**
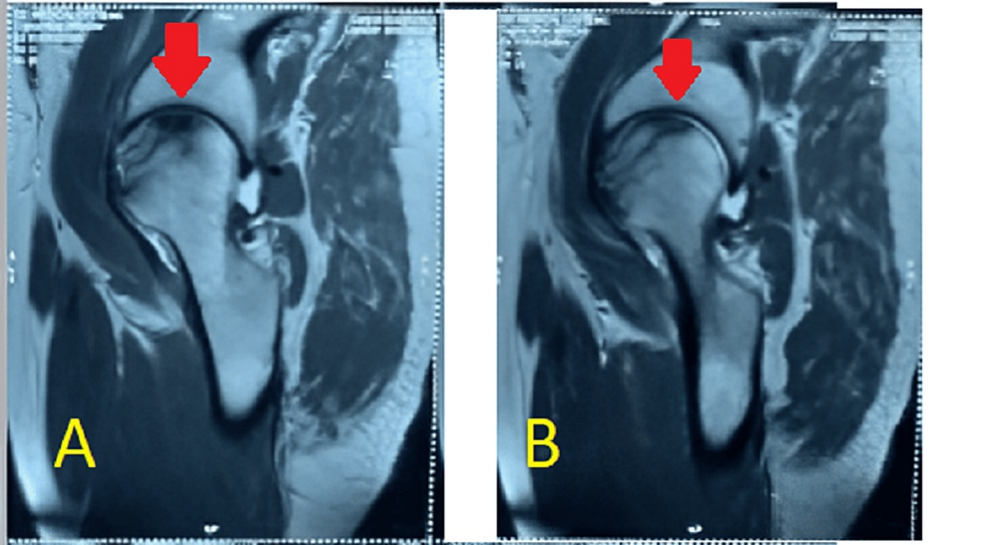
Sagittal section (A) and (B) of MRI, both hips showing destruction on left side The sagittal slices of images A and B show avascular necrosis, or the loss of vascularity, on the left side.

No overt signs of infection or systemic illness were noted. No previous hip surgery or trauma was reported, but the patient did recall a history of occasional heavy alcohol consumption, although he had not consumed alcohol in several years. Anteroposterior and lateral radiographs of the hips were obtained, which revealed signs consistent with bilateral AVN at stage 2, as shown in Figures 1-2. The radiographs demonstrated the presence of cysts in the area of the femoral head. An MRI of both hips was also done, which showed late stage II or early stage III for the left hip and stage II for the right hip, as shown in Figures 3-4. The diagnosis of bilateral hip AVN was confirmed based on clinical presentation and radiographic findings. The MRI report showed modified Ficat and Arlet stage-II/early stage III AVN of the left femoral head involving 75% of the articular surface and modified Ficat and Arlet stage-II avascular necrosis of the right femoral head involving almost 25% of the articular surface.

**Figure 5:**
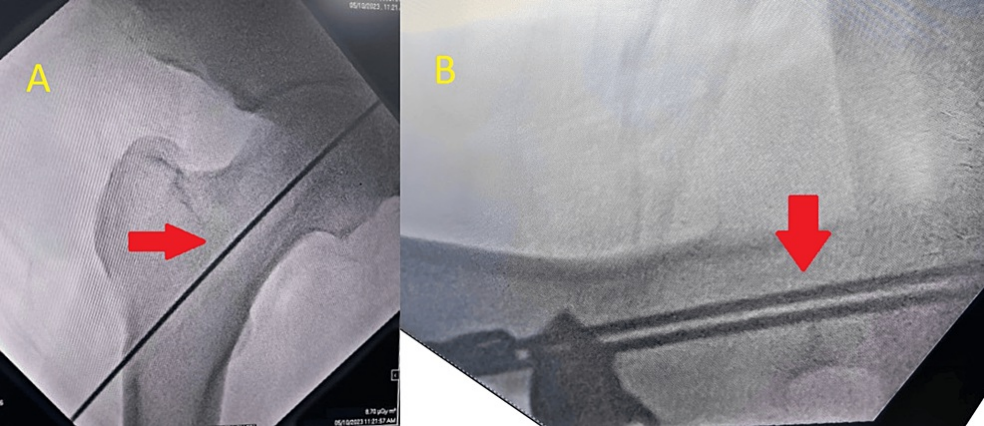
AP (A) and lateral (B) C-arm images of left hip The picture displays two guide wires in the lateral view and one inside in the AP view of the femoral head.

**Figure 6:**
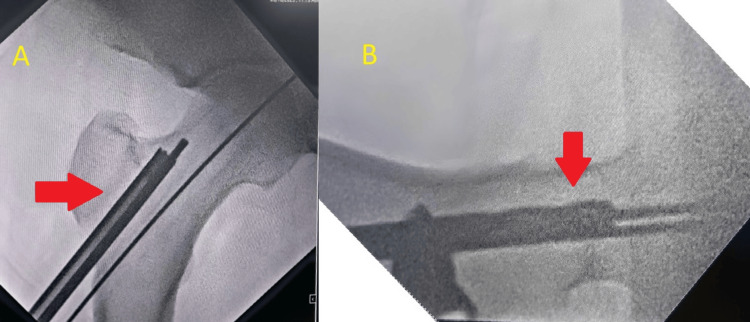
AP (A) and lateral (B) C-arm pictures demonstrating the superior area being triple-reamed

Given the patient's relatively young age and the bilateral involvement of the hips, a comprehensive management plan was developed to address the pain, halt disease progression, and, ideally, promote bone regeneration. The proposed plan included core decompression with reverse bone grafting for both hips. Core decompression is a procedure in which the necrotic bone is removed by reaming with the help of a drill bit. Reverse bone grafting is a procedure that involves the removal of necrotic bone and the replacement of damaged tissue with healthy bone, usually obtained from the patient's own body. In this particular case, we managed the patient with the bone graft that was harvested from the metaphyseal area of the femur during reaming, inserting healthy bone graft into the head of the femur and discarding the sclerotic bone from the head of the femur for each side, starting from the left side as the symptoms were more on the left side. The head of the femur was reamed with a drill bit of 6.5 mm super-centrally and inferno-centrally as shown in Figure 5. The superior one was then triple-reamed up to the size of 10 mm as shown in Figure 6.

**Figure 7:**
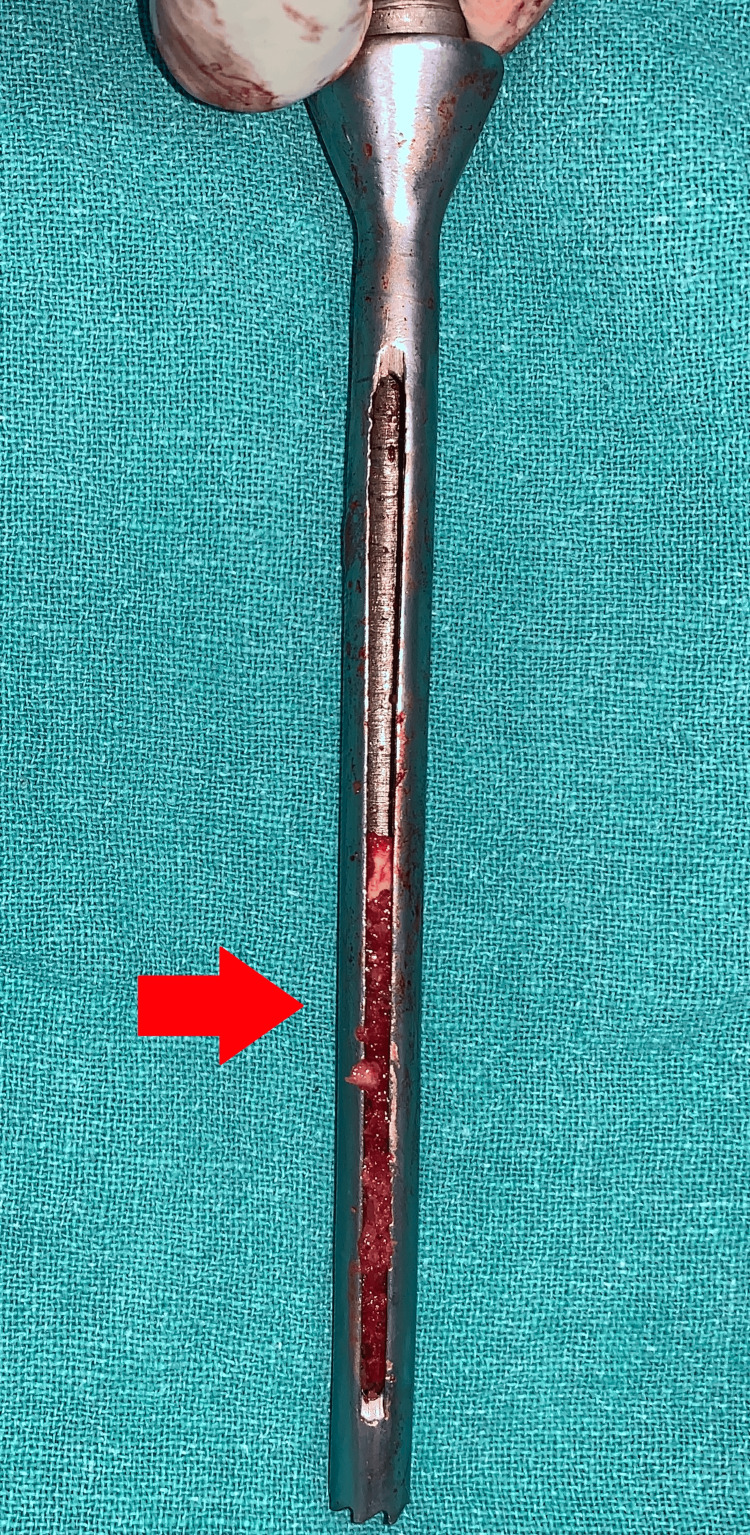
Triple reamer containing bone

**Figure 8:**
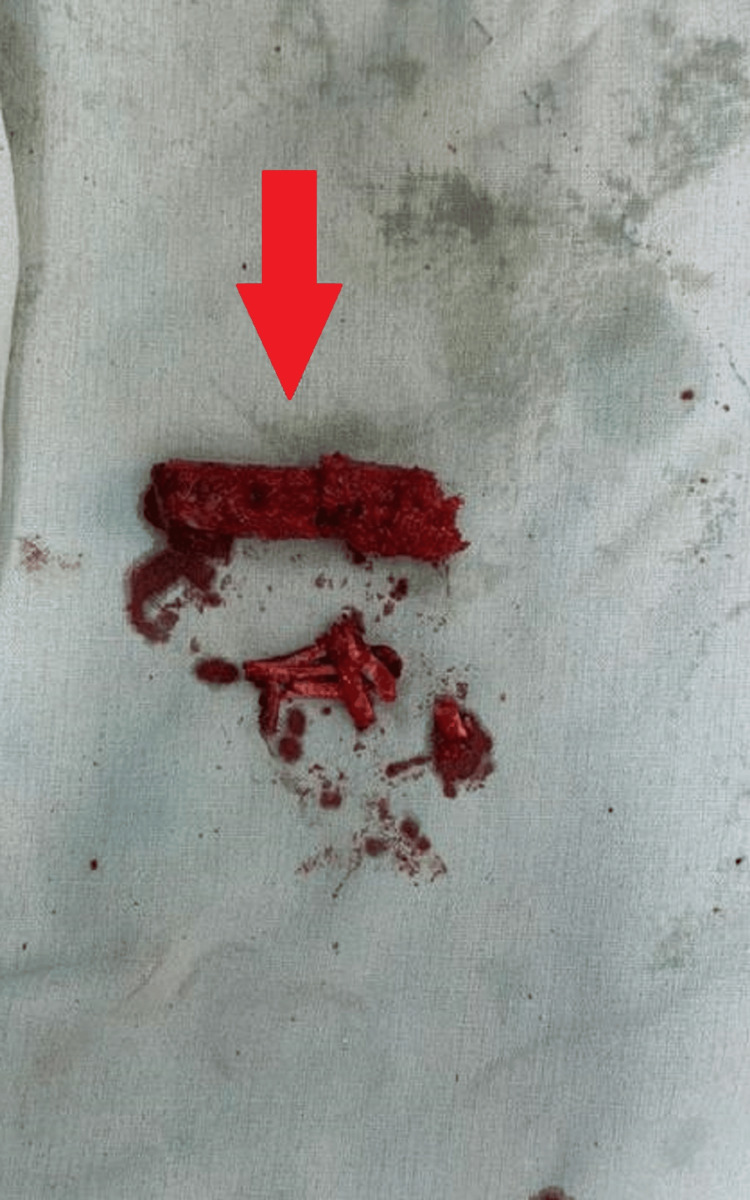
Harvested bone

**Figure 9:**
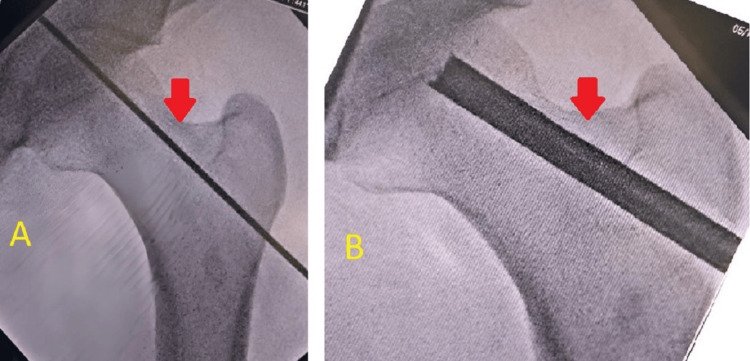
AP (A and B) C-arm showing reaming and triple reaming on the right side

After the reamer was removed, the pieces of bone were present on it, of which, the healthy bone, which appeared red, that was present on the triple reamer (Figure 7) was harvested as seen in Figure 8. The necrotic bone that was present on the tip appeared black and was discarded. The healthy bone was reinserted into the head of the femur followed by BMAC infiltration. A similar procedure was done on the right side with triple reaming up to size 10 mm super-centrally as seen in Figure 9. Similarly, healthy bone was inserted followed by PRP infiltration. BMAC is rich in mesenchymal stem cells, which have the potential to facilitate tissue regeneration. The nucleated cells are approximately four to five times that of whole blood, platelets being three to five times that of bone marrow aspirate. PRP is a concentration of platelets (five to eight times the baseline platelets) and growth factors obtained from the patient's blood, which is known to promote healing and tissue regeneration.

The surgical procedures are tabulated in Table 1.

**Table 1:** Surgical steps followed for the patient BMAC: Bone marrow aspirate concentrate; PRP: Platelet-rich plasma

S. no.	Procedure	Steps
1.	Core decompression (both hips)	The patient underwent core decompression in which the necrotic bone was removed by triple reaming the necrotic area of the femoral head.
2.	Reverse bone grafting (both hips)	The patient underwent reverse bone grafting for both hips in the same surgical session. Necrotic bone was removed from the femoral head, and healthy bone which was harvested from the metaphyseal area of the femur was inserted into the femoral head.
3.	BMAC infiltration (left hip)	For the left hip, following reverse bone grafting, BMAC was obtained from the patient's iliac crest and infiltrated into the affected hip joint to stimulate regeneration and improve tissue health.
4.	PRP Infiltration (right hip)	PRP was made from the patient's blood and injected into the right hip joint after reverse bone grafting to aid with healing and improve tissue regeneration.

**Figure 10:**
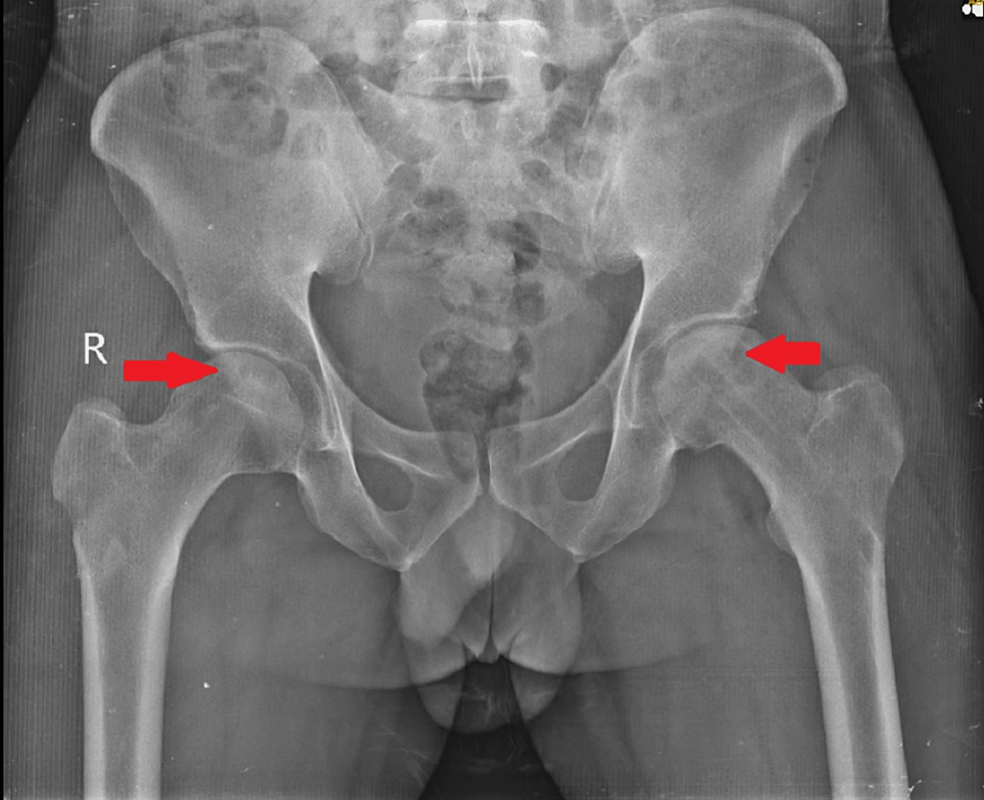
Post-operative AP radiograph of the pelvis showing core decompression with reverse bone grafting

**Figure 11:**
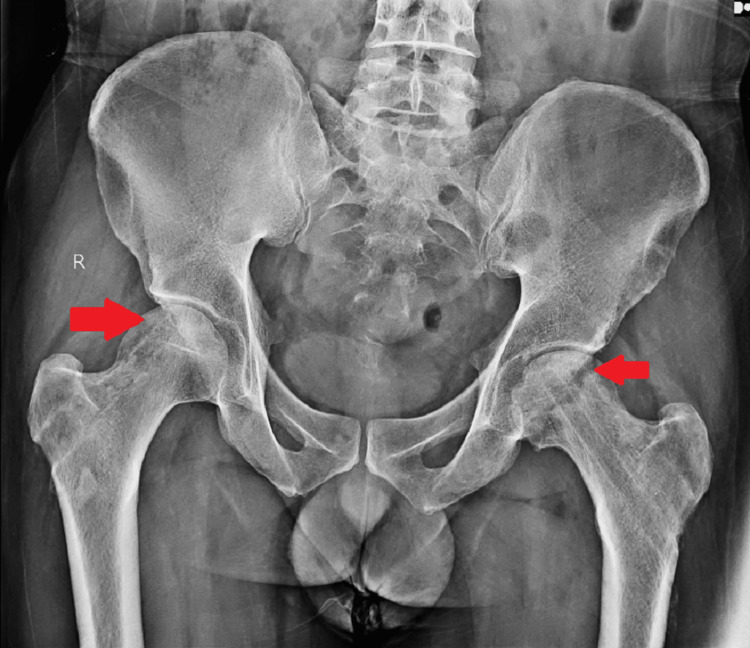
Two-month post-operative AP radiograph of the pelvis Core decompression with reverse bone grafting done and osteoinduction seen on both sides where grafts were inserted.

Post-operatively, the patient had significant pain relief the next day of the procedure, and the hip range of movements was significantly improved. Immediate post-operative movements were: flexion - 70 degrees, external rotation - 35 degrees, internal rotation - 30 degrees, abduction - 40 degrees, and adduction - 30 degrees. The patient was started on anti-osteoporotic treatment in the form of alendronate, calcium, and vitamin D3 supplements to aid in bone healing. A post-operative radiograph was done and was satisfactory as elicited in Figure 10, and a two-month post-operative X-ray was done as shown in Figure 11. The patient had a pain relief of approximately 90% at the follow-up visit after two months.

## Discussion

AVN of the hip in young people is a painful and disabling disorder that can have a long-term impact on mobility and quality of life. Osteonecrosis, another name for AVN, is a condition marked by a progressive deterioration of the hip joint. This condition can cause discomfort, impair movement, and lower quality of life. When both hips are affected at the same time, there are special concerns and problems. Various causative factors from vascular insufficiency include excessive steroid intake, excessive alcohol consumption, trauma, and various coagulation disorders. Medical conditions predisposing to AVN are sickle cell disease, systemic lupus erythematosus (SLE), clotting disorders such as thrombophilia or hypercoagulable states, idiopathic, genetic predisposition, high-dose radiation therapy, certain chemotherapeutic agents, excessive mechanical stresses and joint loading.

This 30-year-old man's case with bilateral AVN emphasizes several important AVN management factors, including diagnosis, surgical intervention, and the application of regenerative medicine procedures to treat the illness as shown in Table 2.

**Table 2:** Management steps of avascular necrosis (AVN)

S. no.	Management steps of AVN	
1	Early diagnosis and assessment	An important aspect of successfully managing AVN is early diagnosis. This instance highlights the value of a comprehensive clinical assessment that includes a radiographic evaluation along with a patient's medical history and physical examination. The precise diagnosis of bilateral hip AVN was made possible by the combination of the radiological results and clinical presentation. Early detection is essential since it may be able to stop the disease's development and maintain joint function.
2	Conservative management	In the early stages, non-surgical or conservative methods can be tried such as medications such as anti-inflammatory drugs, and physical therapy to improve hip joint function and range of motion. In the osteonecrotic lesion site, bisphosphonate reduces osteoclastic activity, which facilitates bone repair. In cases of advanced situations where collapse has already occurred, it delays the necessity for total hip replacement (THR) surgery by preventing the occurrence of subchondral fracture or collapse in the early stages of degenerative hip [10-20]. Activity modifications such as limited weight-bearing and using crutches or other assistive devices.
3	Core decompression	The most often used surgical method for treating AVN is core decompression. Under fluoroscopic supervision, it entails reaming the afflicted region using drill bits of different diameters to remove necrotic bone.
4	Reverse bone grafting	Reverse bone grafting is an established surgical technique for the management of AVN. It involves the removal of necrotic bone tissue from the affected area, which, in this case, was the femoral head, and its replacement with healthy bone tissue. In the case of bilateral AVN, both hips were addressed in a metaphyseal bone of the femur of the same side, which was harvested while reaming in a single surgical session. Reverse bone grafting aims to restore vascularity over the area as well as provide structure support to the femoral head. Numerous surgical teams have successfully carried out free vascularized fibular grafting operations on significant patient groups, with excellent success rates [21- 24].
5	Regenerative medicine techniques	Regenerative medicine techniques including platelet-rich plasma (PRP) infiltration and bone marrow aspirate concentrate (BMAC) infiltration have drawn interest recently as potential complements to conventional therapies. Specifically, the use of stem cell-based treatments has been justified by the idea that they will increase the impact of core decompression by encouraging the production of new bone in AVN [25]. The effectiveness of bone marrow concentrate, the presence of stem cells with osteogenic qualities, the secretion of angiogenic cytokines, which increase angiogenesis and subsequently improve osteogenesis, and the presence of endothelial cell progenitors actively involved in neoangiogenesis from pre-existing capillaries, which can enhance the generation of pericytes and vascular mural cells, are all potentially explained by a variety of factors [25- 27]. The patient in this instance had PRP infiltration in the right hip and BMAC infiltration in the left hip. Mesenchymal stem cells, which may aid in tissue regeneration, are abundant in BMAC. Platelets and growth factors included in PRP can aid in tissue repair and healing. A comparative assessment of these regeneration methods' efficacy was made possible by their selection in a bilateral AVN instance.
6	Comparative outcomes	The outcomes of the hips treated with BMAC infiltration and those treated with PRP infiltration differ significantly, as illustrated in this case study. Following BMAC, there was a more noticeable functional improvement in the left hip coupled with potential signs of bone regeneration. This differentiation underscores the potential benefits of BMAC in stimulating tissue regeneration and restoration.
7	Total hip arthroplasty	If the hip is non-salvageable, or there is extensive damage to the femoral head then the acetabulum along with the femoral head is replaced with prostheses.

A multidisciplinary approach is to be used when addressing AVN in young adults; a multidisciplinary approach is usually required. This case serves as an example of a methodical strategy that involves core decompression, reverse bone grafting, and either BMAC or PRP injection for regenerative methods. The multidisciplinary approach aims to provide long-term solutions that preserve hip function and enhance overall quality of life in addition to addressing the acute symptoms.

## Conclusions

This case highlights the difficulties in treating bilateral AVN in a young adult as well as the possible advantages of reverse bone grafting, core decompression, and regenerative medicine techniques. The necessity of individualized treatment regimens for patients with AVN is suggested by the notable disparities in results between BMAC and PRP infiltration. In these difficult situations, early management and a multidisciplinary approach show promise in maintaining hip function and enhancing quality of life. To fully grasp the regeneration potential of these therapies and improve their use in clinical practice, more research and long-term follow-up studies are necessary.
